# Effects of One-Fifth, One-Third, and One-Half of the Bodyweight Lumbar Traction on the Straight Leg Raise Test and Pain in Prolapsed Intervertebral Disc Patients: A Randomized Controlled Trial

**DOI:** 10.1155/2021/2561502

**Published:** 2021-09-16

**Authors:** Anita Kumari, Nishat Quddus, Prachi Raj Meena, Ahmad H. Alghadir, Masood Khan

**Affiliations:** ^1^Dr. Pradeep Sharma's Pain Management Clinic, New Delhi 110070, India; ^2^Department of Rehabilitation Sciences, Jamia Hamdard, New Delhi 110062, India; ^3^Pandit Deendayal Upadhyaya National Institute for Persons with Physical Disabilities, New Delhi 110002, India; ^4^Rehabilitation Research Chair, College of Applied Medical Sciences, King Saud University, Riyadh 11433, Saudi Arabia

## Abstract

The prolapsed intervertebral disc (PIVD) at the lumbar spine is one of the most common causes of low back pain (LBP) affecting humans worldwide. Lumbar traction is widely used as a part of physiotherapeutic modalities for its treatment; however, reports on its effectiveness and dosage are conflicting. This study is aimed at comparing the acute effects of three traction forces on the straight leg raise (SLR) test and LBP intensity. A total of 45 (age 35.53 yrs., ±3.09) participants with 15 participants in each group were recruited for the study. Participants were divided into groups A, B, and C wherein traction forces equal to one-fifth, one-third, and one-half of their bodyweight were applied, respectively. SLR range of motion (ROM) and pain were examined before and immediately after the application of traction. Significant improvement was observed in SLR ROM in all three groups (*p* < 0.05). However, for pain, significant improvement (*p* < 0.05) was observed only in the group with one-half of bodyweight force. There was no significant difference (*p* > 0.05) between the three groups for both variables. All three forces were equally effective in immediately improving SLR ROM in patients suffering from lumbar PIVD; however, pain improvement was observed with one-half of bodyweight only.

## 1. Introduction

Around 70% of adults suffer from low back pain (LBP) in their lifetime [1]. It is one of the most common health problems affecting humans worldwide [1]. There are many causes for LBP, and one of the most common causes is prolapsed intervertebral discs (PIVD) at the lumbar spine. PIVD is a condition in which the disc bulges or herniates anteriorly or anterolaterally or posteriorly or posterolaterally. Posterior or posterolateral disc prolapse is common and may press the nerve roots in the spinal canal [2]. As per estimation, around 10% of LBP episodes are associated with nerve root involvement [3, 4]. Nerve root involvement is associated with more severe symptoms, more risk of chronicity, more work absence, and higher healthcare costs such as direct treatment costs, disability benefits, and insurance [5, 6]. Both surgical and nonsurgical treatments are available for disc prolapse, but nonsurgical treatment is recommended initially in most cases. Physical therapy is one of the main constituents of nonsurgical management for such patients. Physical therapy treatment for PIVD includes mobilization exercise, spinal manipulation, strengthening exercises, mechanical traction, and other electrotherapeutic modalities.

The mechanism of action of mechanical lumbar traction is not well defined, but it is proposed that traction separates the vertebral bodies, decreasing the compressive forces on herniated discs. Vertebral separation also enlarges the intervertebral foramen, which decreases the nerve root compression because now more space is available for the disc and nerves. It also puts tension on the spinal ligaments, which helps the discs to return to their normal position. New studies have reported that herniated mass size decreases with segmental traction [7, 8].

Some studies support the use of traction to treat disc-related symptoms and while others do not. For example, a systematic review by Clarke et al. [9] concluded that intermittent or continuous traction as a single treatment cannot be recommended for treatment of LBP with or without sciatica. A systematic review by Wegner et al. concluded that traction, whether given alone or in combination with other interventions, has either little or no effect on pain, functional status, and return to work in patients with LBP [10]. A recent systematic review and meta-analysis by Vanti et al. [11] reported that supine mechanical traction has short-term effects on pain and disability if it is provided along with other physical therapy interventions for lumbar radiculopathy patients. Thus, there is great conflict regarding the effectiveness of lumbar traction. There is also no consensus in the literature regarding the dosage of traction. One study was performed by Meszaros et al. [12] which reported that 30% and 60% of bodyweight traction forces were effective in increasing straight leg raise (SLR) measurements in patients with positive SLR below 45°. A study by Alrwaily et al. [13] reported that there is wide variability in the traction parameters among the RCTs of lumbar traction. Therefore, no particular dosage is prescribed for treatment. Earlier studies reported the use of large bodyweight traction forces to cause vertebral separation, such as Cyriax reported that a static traction force of 120 lbs for 15 minutes is necessary to cause vertebral separation [14]. As per Judovich, a force equal to at least one-half of the body is required to cause therapeutic effects in the lumbar spine [15]. Others have proposed smaller forces, such as Crisp et al. [16] who believed that 40 to 80 lbs, applied for 15-20 minutes, are sufficient. Therefore, one study was warranted that compares the effectiveness of different traction forces. Thus, this study is aimed at comparing the acute effects of three different traction forces (one-fifth, one-third, and one-half of bodyweight) applied in Fowler's position, on SLR range of motion (ROM) and LBP intensity in patients with lumbar PIVD.

## 2. Materials and Methods

### 2.1. Participants

**Figure 1:**
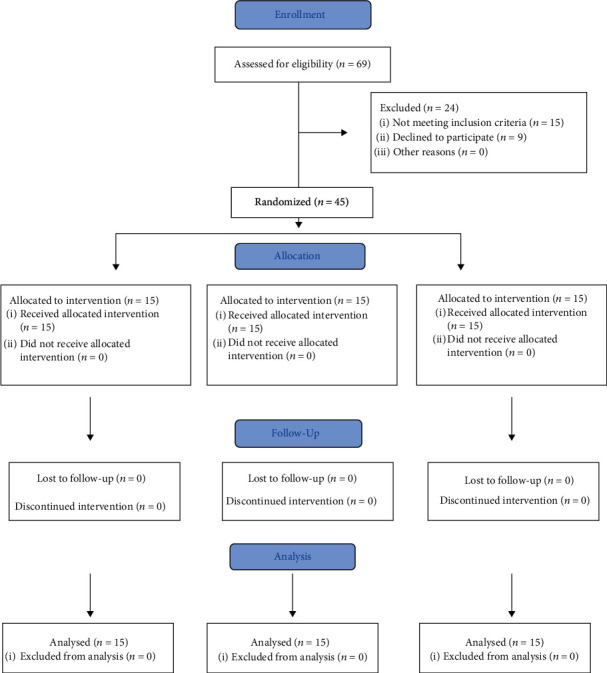
Consolidated Standards of Reporting Trials (CONSORT) flow chart of the study showing recruitment of participants.

Similar previous study performed by Meszaros et al. [12] recruited ten subjects; therefore, convenient sampling was performed and forty-five participants (age 35.53 yrs., ±3.09) (30 males and 15 females) with fifteen participants in each group were selected for the study (Table 1, Figure 1). Selected participants had low back pain for less than three months, a positive unilateral SLR test which included reports of low back and leg pain or paraesthesia below 45° hip flexion. They also had to have at least one additional neurological sign including diminished Achilles/hamstring tendon reflex tested in the supine position or hypoesthesia in any of the L4-S2 dermatomes or muscle weakness in any of the L4-S2 myotome. Radiologists diagnosed the posterior or posterolateral PIVD using MRI of the lumbar spine at either L4-L5 or L5-S1 level or both. Participants who were currently receiving or had received physical therapy treatment in the last three months were excluded from the study. In addition, participants having malignancy, tuberculosis, osteoporosis, osteomyelitis of the vertebral column, cord compression, pregnancy, or joint hypermobility were excluded from the study. It also excluded those participants that could not tolerate traction treatment secondary to severe acute pain. The study was conducted according to the guidelines of the Declaration of Helsinki and approved by the ethical committee of the Institutional Review Board (protocol code: RRC-2019-29 and date of approval 5^th^ December 2019). This study was conducted in a university medical hospital. This study has been registered prospectively before the recruitment of participants in http://clinicaltrial.gov (ID: NCT04728685).

### 2.2. Study Design

A three-arm parallel pretest-posttest experimental research design with random allocation (allocation ratio 1 : 1 : 1) of participants into three groups was used. Concealed allocation of participants was performed using the lottery method and the website http://www.randomization.com by a researcher who was not involved in the recruitment or assessment of the participants. Forty-five chits with one number from 1 to 45 written over them were placed in a box. Once a participant was deemed fit for the study, the examiner picked one chit from the box. That number was allocated to that participant. Random permutation of integers from 1 to 45 was generated for the three groups using the website http://www.randomization.com. Then accordingly, participants were allocated into either of the three groups (groups A, B, or C). One-fifth of bodyweight (in group A), one-third of bodyweight (in group B), and one-half of bodyweight (in group C) traction forces were applied. The participants and the outcome assessor, i.e., the examiner who measured LBP and SLR, were kept blinded to the allocation. Before the application of any intervention, the risks and benefits of the study were discussed with the participants, and informed consent was obtained. LBP intensity and SLR ROM were dependent variables, and traction therapy was an independent variable. The LBP intensity and SLR ROM were measured using a visual analog scale (VAS) and a goniometer, respectively.

### 2.3. Outcome Measures

Outcome measures include (a) SLR ROM and (b) VAS scores. (a) SLR test is among the commonly used tests to diagnose lower lumbar radiculopathy. It has high sensitivity (91%) [17] in detecting prolapsed intervertebral discs at the lumbar spine. (b) VAS is a valid, reliable, and one of the most frequently used pain outcome measures [18]. This scale consists of a 10 cm long line with integers from 0 to 10 written at an interval of 1 cm, with 0 at one end and 10 at another end. Here, 0 indicates “no pain” and 10 indicates “worst pain imaginable.” Participants were asked to mark the line according to the pain they were perceiving [18].

### 2.4. Instrumentation

- Traction unit (Huntleigh Akron ATP9)
- Footstool
- Goniometer—universal full circle
- VAS
- Weighing machine

### 2.5. Study Protocol

The study consisted of three phases: preintervention assessment, intervention, and postintervention assessment.

#### 2.5.1. Preintervention Assessment

Before applying the intervention, a neurological examination was conducted that included sensation, reflex, and muscle strength testing of the lower extremities. Participant's demographic data was collected including their bodyweight in kg. Bodyweight was measured using the weighing machine. One-fifth of bodyweight (participants in group A), one-third of bodyweight (participants in group B), and one-half of bodyweight (participants in group C) were calculated. Then, passive SLR ROM of the ipsilateral lower extremity and low back pain intensity on the VAS were noted. These outcome variables were measured by the same examiner to avoid any subjective variability in measurements. Participants were asked to report the level of LBP they had on a scale of 10 points where 0 indicated no pain and 10 indicated the maximum pain they have ever felt.

SLR ROM measurement: For SLR ROM measurement, participants lied supine on the traction table, their ipsilateral lower extremity was passively raised by an assistant from the couch with their ankle in a neutral position, and knee completely extended. When participants complained of pain in the back or leg or paresthesia in the lower limb, then limb elevation was stopped and that degree of hip flexion was noted with a goniometer by the examiner. The goniometer's fulcrum was placed over the greater trochanter, its stationary arm parallel to the table, and the moving arm along the lateral midline of the thigh [12]. A total of three readings were taken, and an average of these three readings was taken for data analysis.

#### 2.5.2. Intervention

Lumbar traction was applied in Fowler's position. Participants who were lying on the traction table were fitted with a thoracic and pelvic harness. The hips and knees of participants were flexed to 90° by keeping a padded footstool beneath both legs; thus, the traction sling made an angle of pull of 18° [19]. Participants in group A received one-fifth of bodyweight, in group B one-third of bodyweight, and in group C one-half of their bodyweight traction force. It was static traction that was applied for 10 minutes on a split table.

#### 2.5.3. Postintervention Assessment

Passive SLR ROM and pain intensity on the VAS were again noted immediately after traction by the same examiner and assistant.

### 2.6. Data Analysis

Forty-five participants were analyzed for the study. All data analysis was performed using SPSS statistical software version 26 (SPSS Inc., Chicago, IL, USA). The Shapiro-Wilk test of normality was used to assess the normal distribution of demographic (age, height, weight, and BMI) and baseline variables (SLR and VAS) data. The Mann-Whitney test was used to compare the baseline value of dependent variables (SLR and VAS) data in between all three groups. The Mann-Whitney test was performed to compare demographic data and baseline values of variables. The Shapiro-Wilk test of normality revealed a normal distribution of all data except for BMI in group B and baseline VAS data for all three groups. Therefore, nonparametric tests were used for further within- and between-group comparisons. For within-group analysis, the Wilcoxon signed-rank test was performed to assess change in dependent variables data after the intervention. For between-group analysis, the Mann-Whitney test was performed to compare dependent variables between all three groups. The confidence interval was set at 95% with a *p* value < 0.05 considered as significant.

## 3. Results

Mean ± SD values of dependent variables (SLR and VAS) are presented in Table 2. The Shapiro-Wilk test of normality showed a normal distribution for all demographic (age, height, weight; df = 15, *p* > 0.05) and independent variables data (SLR and VAS; df = 15, *p* > 0.05) except for BMI (df = 15, *p* < 0.05) in group B and baseline VAS values (df = 15, *p* < 0.05) in all three groups. The Mann-Whitney test performed to compare demographic (Table 3) and baseline values of independent variables (SLR and VAS) did not show any significant differences between all three groups; thus, all three groups were similar to each other in terms of participants' age, height, weight, BMI, SLR degree, and their VAS scores; thus, they were comparable.

### 3.1. Within-Group Analysis

**Figure 2:**
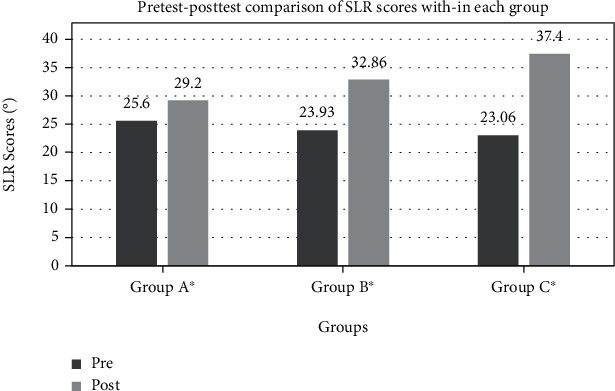
Pretest-posttest within-group comparison for the scores of outcome SLR. ^∗^Mean differences are significant (*p* < 0.05).

**Figure 3:**
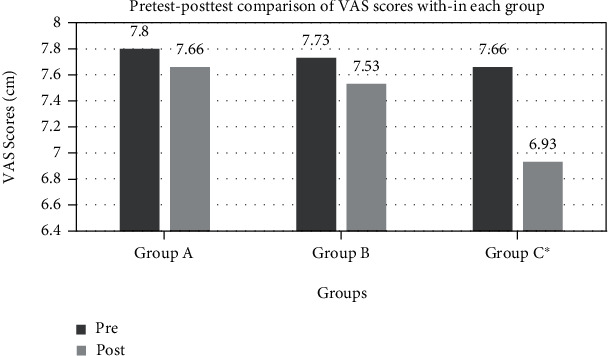
Pretest-posttest within-group comparison for the scores of outcome VAS. ^∗^Mean differences are significant (*p* < 0.05).

The Wilcoxon signed-rank test revealed a significant improvement in SLR degrees in all three groups (*p* < 0.05) (Table 4, Figure 2). For the VAS, a significant improvement was observed in group C (*p* < 0.05); however, in groups A and B, the change in VAS was insignificant (*p* > 0.05) (Figure 3).

### 3.2. Between-Group Analysis

The Mann-Whitney test revealed no significant differences between groups A, B, and C (*p* > 0.05) for both independent variables (SLR and VAS) (Table 5).

## 4. Discussion

The present study is aimed at comparing the immediate effects of one-fifth, one-third, and one-half of the bodyweight traction forces on the SLR ROM and LBP in lumbar PIVD patients. The results showed that there was a significant improvement in SLR degree with all three intensities of traction force. However, for LBP, only the groups where one-half of bodyweight traction was applied showed improvement. All three traction forces increased the SLR degrees, but no difference was observed between the three forces, which showed that all three forces are equally effective in improving SLR ROM.

Previously, not many studies were performed that compared the acute effects of different intensities of traction forces on SLR ROM and LBP intensity. The participants recruited in this study were required to have back pain along with a positive SLR test and diagnosed with PIVD at the lower lumbar level through MRI. SLR test was chosen as it is the most appropriate indicator of nerve root irritation and the most widely used test in clinical settings [12]. If there is compression on the nerve root because of disc prolapse, then the nerve cannot move freely; thus, the pain-sensitive dural sheath of the nerve will restrict the passive SLR ROM [12]. Surgical findings in the case of limited SLR ROM showed that the restriction in the mobility of the nerve is related to root compression because of the herniated disc [20–22].

Three different intensities of traction forces were compared in the present study because there is no consistency in the literature regarding the forces to use for therapeutic traction. Studies have reported that a traction force of ≥25% (one-fourth) of bodyweight can separate the lumbar vertebrae [10, 23], but it is not specified what range of a lumbar traction force is optimal or most therapeutic for PIVD patients.

Fowler's position was chosen for the study because the posterior soft collagenous tissues are slack in the neutral position of the lumbar spine [24, 25]. Therefore, if a traction force is applied in a supine lying position (neutral lumbar spine), a considerable force will be spent just to take up that soft tissue slack. However, if Fowler's position is held by the patient, then the lumbar spine will go into flexion, the posterior fibers will be stretched, and thus, the slack will be taken up. Therefore, in Fowler's position, less traction force is required to stretch the posterior tissues [19]. Also, the angle of pull on the pelvic traction harness was set to 18°, because most of the previous authors recommended this for optimal results [19].

In the present study, an improvement in the SLR ROM was observed even with the smallest traction force of one-fifth of bodyweight. The possible mechanisms behind this improvement could be explained: first, axial traction elongates the vertebral column, thus opening up the intervertebral disc space (IVDS) [26–28]. This causes a negative suction pressure inside the IVDS due to which the herniated disc slides back [29]. Now because of the sliding back of the herniated disc, the normal slack in the neuromeningeal sheath may be restored, which may allow an increase in movement of the lower extremity during the SLR test. Another mechanism proposed is that traction causes a lessening of muscle spasms, thus causing relaxation of paraspinal musculatures. This may cause a reduction in spasmodic compressive forces in the vertebral column that may increase IVDS [30]. And last is the retractile force induced by the posterior longitudinal ligaments: It is believed that traction causes an increase in tension in the posterior longitudinal ligaments, because of which a retractile force is generated that pushes the herniated disc mass back into IVDS, reducing the herniated disc size [19]. However, this mechanism is likely to operate when the prolapsed disc material has not extended beyond the annulus fibrosus and posterior longitudinal ligaments.

In our study, small traction forces (one-fifth and one-third of bodyweight) did not cause any change in LBP; however, one-half of bodyweight force resulted in LBP reduction by 9.53%. This could be because traction causes the opening of the intervertebral foramen [19, 31], and thus, the pressure on the impinged neural structures is lessened [32]. This may reduce pain. Moreover, traction causes stretching of paraspinal muscles, facet joints, ligaments, and discs [33]. It is hypothesized that mechanoreceptors present in these structures are stimulated because of stretching, which may cause inhibition of pain impulses [34–36]. It is also proposed that stretching of ligamentous and osseous structures may improve nutrition to local impinged neural and ligamentous structures, thus causing reduced pain transmission [35]. Traction is also claimed to cause relaxation of paraspinal muscles, thus breaking the pain-spasm cycle [30] and relieving the pain.

Since all three (one-fifth, one-third, and one-half of the bodyweight) forces had statistically the same effects on SLR ROM, therefore, the smallest force, i.e., one-fifth of bodyweight, should be used for treatment. Because it is reported that general axial traction may put high stresses on the posterior fibers of the annulus fibrosus and traction forces greater than the optimal amount can even cause rupture of these fibers [37].

The present study had several limitations as well. The sample size in the present study was small. To generalize the result, much larger sample size is needed. Another limitation is that traction therapy is not used alone to treat disc-related problems. Usually, in clinical settings, traction is given along with other electrotherapeutic modalities, exercises, mobilization techniques, etc. Therefore, studying the effects of traction in isolation will not have much practical application. Further research is needed that can examine the therapeutic effects of a rehabilitation protocol that includes mechanical traction along with other physical therapy modalities. In addition, the present study examined the immediate effects only; however, it is unknown for how long the improvement in SLR ROM or LBP will persist. Therefore, more studies are needed that examine the long-term effects of traction to know if the improvement obtained is long-lasting or symptom relapse. This study examined the effects of traction when it is applied once only. Usually, for the complete resolution of symptoms in such patients, a rehabilitation program lasting from several days to several months is required. Therefore, studying the effects of traction when applied only once will not provide a sound clinical implication. The present study did not use any soft tissue imaging technique like magnetic resonance imaging (MRI) to examine the effects of traction on intervertebral disc space or intervertebral foremen. Use of such imaging technique before, during, and after traction therapy in future research may substantiate the findings of previous studies that reported an opening in intervertebral foremen due to traction [19, 31].

## 5. Conclusions

One-fifth, one-third, and one-half of bodyweight traction forces are equally effective in improving the SLR ROM in patients suffering from lumbar PIVD. LBP improvement was observed with one-half of bodyweight traction only.

## Figures and Tables

**Table 1:** Respondent's demographic data, mean ± SD, and *p* values for the Shapiro-Wilk test of normality.

	Group A	*p* value	Group B	*p* value	Group C	*p* value
Age (years)	34.93 ± 3.15	0.16	35.73 ± 2.93	0.54	35.93 ± 3.30	0.11
Height (cm)	165.73 ± 6.76	0.30	166.13 ± 6.78	0.07	166.66 ± 7.55	0.27
Weight (kg)	64.80 ± 12.06	0.53	63.33 ± 11.04	0.29	67.26 ± 12.22	0.96
BMI (kg/m^2^)	23.48 ± 3.61	0.06	22.84 ± 3.08	0.01^∗^	24.02 ± 2.74	0.94
PreSLR (degrees)	25.60 ± 7.02	0.87	23.93 ± 9.16	0.85	23.06 ± 11.06	0.15
PreVAS (cm)	7.80 ± 0.56	≤0.01^∗^	7.73 ± 0.70	≤0.01^∗^	7.66 ± 0.61	≤0.01^∗^

^∗^Significant. BMI: body mass index; SLR: straight leg raise; VAS: visual analog scale.

**Table 2:** SLR and VAS; mean ± SD values at baseline (pre) and after intervention (post).

	Group A	Group B	Group C
PreSLR (degree)	25.60 ± 7.02	23.93 ± 9.16	23.06 ± 11.06
PostSLR (degree)	29.20 ± 8.58	32.86 ± 10.07	37.40 ± 14.95
PreVAS (cm)	7.80 ± 0.56	7.73 ± 0.70	7.66 ± 0.61
PostVAS (cm)	7.66 ± 0.81	7.53 ± 0.74	6.93 ± 1.57

SLR: straight leg raise; VAS: visual analog scale.

**Table 3:** Between-group comparison of demographic data (Mann-Whitney test).

	Groups A and B (*p* values)	Groups A and C (*p* values)	Groups B and C (*p* values)
Age (years)	0.45	0.41	0.83
Height (cm)	0.86	0.67	0.72
Weight (kg)	0.83	0.63	0.28
BMI (kg/m^2^)	0.59	0.59	0.11

BMI: body mass index.

**Table 4:** Within-group (Wilcoxon signed-rank test) comparison for dependent variables in both groups.

	Group A		Group B		Group C	
	*Z*	*p* value	*Z*	*p* value	*Z*	*p* value
PostSLR-PreSLR	-2.937	≤0.01^∗^	-3.41	≤0.01^∗^	-3.41	≤0.01^∗^
PostVAS-PreVAS	-1.41	0.15	-1.73	0.08	-2.23	0.02^∗^

^∗^Significant. SLR: straight leg raise; VAS: visual analog scale.

**Table 5:** Between-group (Mann-Whitney test) comparison of dependent variables; mean difference ± SD.

	Groups A and B (*p* value)	Groups A and C (*p* value)	Groups B and C (*p* value)
PreSLR	0.46	0.44	0.77
PostSLR	0.28	0.12	0.61
PreVAS	0.69	0.50	0.83
PostVAS	0.45	0.16	0.41

SD: standard deviation; SLR: straight leg raise; VAS: visual analog scale.

## Data Availability

The data associated with the paper has been uploaded in supplementary data (available here).

## Supplementary Materials

Supplementary Materials The data associated with the paper has been uploaded in supplementary data.
