# Supplementary material for: Effects of One-Fifth, One-Third, and One-Half of the Bodyweight Lumbar Traction on the Straight Leg Raise Test and Pain in Prolapsed Intervertebral Disc Patients: A Randomized Controlled Trial
Source: Biomed Res Int. 2021 Sep 16;2021:2561502. doi: 10.1155/2021/2561502 (PMC8463178; doi:10.1155/2021/2561502)
Supplement: Supplementary Materials — The data associated with the paper has been uploaded in supplementary data. [file 2561502.f1.docx]

| Groups | Subjects S.No. | Age (Yrs) | Sex | Height (cm) | Height (m) | Height (m^2^) | BMI | Weight (Kg) | Pre SLR 1 | Pre SLR 2 | Pre SLR 3 | Avg Pre SLR | Post SLR | Pre VAS | Post VAS |
| --- | --- | --- | --- | --- | --- | --- | --- | --- | --- | --- | --- | --- | --- | --- | --- |
| A | 1 | 37 | F | 153 | 1.53 | 2.3409 | 20.0777479 | 47 | 18 | 22 | 20 | 20 | 20 | 8 | 8 |
| A | 2 | 33 | M | 172 | 1.72 | 2.9584 | 21.9713359 | 65 | 23 | 25 | 30 | 26 | 30 | 7 | 7 |
| A | 3 | 40 | M | 170 | 1.7 | 2.89 | 24.9134948 | 72 | 12 | 11 | 13 | 12 | 14 | 8 | 8 |
| A | 4 | 39 | F | 160 | 1.6 | 2.56 | 32.03125 | 82 | 34 | 34 | 31 | 33 | 39 | 8 | 8 |
| A | 5 | 36 | M | 168 | 1.68 | 2.8224 | 22.3214286 | 63 | 17 | 18 | 19 | 18 | 18 | 8 | 8 |
| A | 6 | 31 | M | 165 | 1.65 | 2.7225 | 19.8347107 | 54 | 23 | 23 | 23 | 23 | 23 | 9 | 9 |
| A | 7 | 35 | F | 154 | 1.54 | 2.3716 | 20.2395008 | 48 | 32 | 30 | 31 | 31 | 34 | 8 | 8 |
| A | 8 | 32 | M | 170 | 1.7 | 2.89 | 27.3356401 | 79 | 29 | 25 | 27 | 27 | 34 | 8 | 8 |
| A | 9 | 40 | M | 175 | 1.75 | 3.0625 | 27.4285714 | 84 | 25 | 25 | 25 | 25 | 26 | 7 | 7 |
| A | 10 | 37 | M | 169 | 1.69 | 2.8561 | 21.7079234 | 62 | 22 | 25 | 22 | 23 | 35 | 7 | 6 |
| A | 11 | 35 | M | 160 | 1.6 | 2.56 | 19.921875 | 51 | 27 | 30 | 27 | 28 | 33 | 7 | 6 |
| A | 12 | 31 | F | 162 | 1.62 | 2.6244 | 25.5296449 | 67 | 35 | 33 | 40 | 36 | 37 | 8 | 8 |
| A | 13 | 34 | M | 171 | 1.71 | 2.9241 | 19.835163 | 58 | 32 | 33 | 31 | 32 | 42 | 8 | 8 |
| A | 14 | 32 | F | 164 | 1.64 | 2.6896 | 23.4235574 | 63 | 36 | 32 | 34 | 34 | 34 | 8 | 8 |
| A | 15 | 32 | M | 173 | 1.73 | 2.9929 | 25.7275552 | 77 | 17 | 16 | 15 | 16 | 19 | 8 | 8 |
| B | 16 | 38 | M | 167 | 1.67 | 2.7889 | 22.9481157 | 64 | 31 | 33 | 32 | 32 | 35 | 8 | 8 |
| B | 17 | 36 | F | 155 | 1.55 | 2.4025 | 21.6441207 | 52 | 12 | 10 | 8 | 10 | 20 | 8 | 8 |
| B | 18 | 35 | M | 173 | 1.73 | 2.9929 | 22.3863143 | 67 | 35 | 33 | 37 | 35 | 47 | 7 | 6 |
| B | 19 | 40 | M | 168 | 1.68 | 2.8224 | 29.7619048 | 84 | 28 | 28 | 28 | 28 | 34 | 7 | 7 |
| B | 20 | 31 | M | 172 | 1.72 | 2.9584 | 21.9713359 | 65 | 23 | 21 | 16 | 20 | 25 | 8 | 8 |
| B | 21 | 38 | M | 176 | 1.76 | 3.0976 | 21.3068182 | 66 | 16 | 15 | 17 | 16 | 25 | 8 | 7 |
| B | 22 | 32 | F | 155 | 1.55 | 2.4025 | 21.6441207 | 52 | 23 | 24 | 25 | 24 | 29 | 7 | 7 |
| B | 23 | 34 | M | 166 | 1.66 | 2.7556 | 19.2335607 | 53 | 24 | 22 | 23 | 23 | 40 | 7 | 7 |
| B | 24 | 33 | F | 156 | 1.56 | 2.4336 | 19.7238659 | 48 | 13 | 17 | 15 | 15 | 22 | 9 | 8 |
| B | 25 | 39 | M | 171 | 1.71 | 2.9241 | 21.2031052 | 62 | 26 | 25 | 27 | 26 | 39 | 8 | 8 |
| B | 26 | 40 | M | 168 | 1.68 | 2.8224 | 25.1558957 | 71 | 35 | 37 | 30 | 34 | 42 | 8 | 8 |
| B | 27 | 36 | F | 169 | 1.69 | 2.8561 | 29.0606071 | 83 | 20 | 24 | 22 | 22 | 38 | 7 | 7 |
| B | 28 | 32 | M | 166 | 1.66 | 2.7556 | 21.0480476 | 58 | 15 | 19 | 17 | 17 | 25 | 8 | 8 |
| B | 29 | 37 | F | 171 | 1.71 | 2.9241 | 24.6229609 | 72 | 11 | 14 | 17 | 14 | 20 | 9 | 9 |
| B | 30 | 35 | M | 159 | 1.59 | 2.5281 | 20.9643606 | 53 | 41 | 44 | 44 | 43 | 52 | 7 | 7 |
| C | 31 | 36 | F | 157 | 1.57 | 2.4649 | 21.0961905 | 52 | 36 | 35 | 34 | 35 | 43 | 7 | 3 |
| C | 32 | 37 | F | 163 | 1.63 | 2.6569 | 27.099251 | 72 | 41 | 40 | 39 | 40 | 65 | 8 | 7 |
| C | 33 | 32 | M | 176 | 1.76 | 3.0976 | 24.535124 | 76 | 16 | 23 | 27 | 22 | 37 | 7 | 5 |
| C | 34 | 39 | F | 157 | 1.57 | 2.4649 | 21.9075825 | 54 | 36 | 33 | 36 | 35 | 52 | 7 | 5 |
| C | 35 | 40 | M | 177 | 1.77 | 3.1329 | 26.8122187 | 84 | 14 | 11 | 11 | 12 | 20 | 8 | 8 |
| C | 36 | 36 | M | 175 | 1.75 | 3.0625 | 24.8163265 | 76 | 23 | 22 | 21 | 22 | 34 | 9 | 9 |
| C | 37 | 40 | M | 170 | 1.7 | 2.89 | 23.183391 | 67 | 10 | 10 | 10 | 10 | 18 | 8 | 8 |
| C | 38 | 31 | M | 167 | 1.67 | 2.7889 | 21.5138585 | 60 | 13 | 11 | 12 | 12 | 18 | 8 | 8 |
| C | 39 | 38 | M | 171 | 1.71 | 2.9241 | 30.09473 | 88 | 9 | 7 | 8 | 8 | 22 | 8 | 8 |
| C | 40 | 31 | M | 163 | 1.63 | 2.6569 | 23.3354661 | 62 | 26 | 20 | 20 | 22 | 35 | 8 | 8 |
| C | 41 | 38 | M | 173 | 1.73 | 2.9929 | 26.0616793 | 78 | 38 | 40 | 42 | 40 | 60 | 7 | 6 |
| C | 42 | 35 | F | 154 | 1.54 | 2.3716 | 19.3961882 | 46 | 11 | 11 | 8 | 10 | 35 | 8 | 7 |
| C | 43 | 32 | M | 169 | 1.69 | 2.8561 | 23.1084346 | 66 | 23 | 33 | 25 | 27 | 34 | 7 | 7 |
| C | 44 | 40 | M | 170 | 1.7 | 2.89 | 24.9134948 | 72 | 28 | 27 | 29 | 28 | 54 | 7 | 7 |
| C | 45 | 34 | F | 158 | 1.58 | 2.4964 | 22.4323025 | 56 | 21 | 28 | 20 | 23 | 34 | 8 | 8 |
